# Effects of Long-Term Exposure to L-Band High-Power Microwave on the Brain Function of Male Mice

**DOI:** 10.1155/2021/2237370

**Published:** 2021-09-04

**Authors:** Yanyun Lin, Peng Gao, Yichen Guo, Qin Chen, Haiyang Lang, Qiyan Guo, Xia Miao, Jing Li, Lihua Zeng, Guozhen Guo

**Affiliations:** ^1^Department of Radiation Medicine and Protection, Faculty of Preventive Medicine, Airforce Medical University, Xi'an, Shaanxi 710032, China; ^2^Department of Biological Science and Bioengineering, Key Laboratory of Biomedical Information Engineering of the Ministry of Education, School of Life Science and Technology, Xi'an Jiaotong University, Xi'an, Shaanxi 710049, China; ^3^Ministry of Education Key Lab of Hazard Assessment and Control in Special Operational Environment, Xi'an, Shaanxi 710032, China; ^4^Xianghu Institute for Applied Sciences, Hangzhou, Zhejiang 311200, China; ^5^Department of Infection Prevention and Control, General Hospital of Western Military Region, Chengdu, Sichuan 610083, China

## Abstract

Currently, the impact of electromagnetic field (EMF) exposure on the nervous system is an increasingly arousing public concern. The present study was designed to explore the effects of continuous long-term exposure to L-band high-power microwave (L-HPM) on brain function and related mechanisms. Forty-eight male Institute of Cancer Research (ICR) mice were exposed to L-HPM at various power densities (0.5, 1.0, and 1.5 W/m^2^) and the brain function was examined at different time periods after exposure. The morphology of the brain was examined by hematoxylin-eosin (HE) and deoxynucleotidyl transferase-mediated dUTP nick-end labeling (TUNEL) staining. Furthermore, cholinergic markers, oxidative stress markers, and the expression of c-fos were evaluated to identify a “potential” mechanism. The results showed that exposure to L-HPM at 1.5 W/m^2^ can cause generalized injuries in the hippocampus (CA1 and CA3) and cerebral cortex (the first somatosensory cortex) of mice, including cell apoptosis, cholinergic dysfunction, and oxidative damage. Moreover, the deleterious effects were closely related to the power density and exposure time, indicating that long-term and high-power density exposure may be detrimental to the nervous system.

## 1. Introduction

Since regular radio broadcasts started in the 1920s, exposure to human-made electromagnetic fields (EMFs) has steadily increased. Nowadays, radio waves come not only from radios but also from a variety of other sources, such as navigation and communication systems, as well as high-voltage transmission and transformation systems. Consequently, a very large fraction of the global population is exposed to EMFs. Unfortunately, the mainstream view in academia is that long-term and high-intensity EMF exposure may disrupt the homeostasis of biological systems and harm human health [1–3]. Although EMF technologies have brought a lot of convenience to human life, there is still insufficient knowledge on the biological effects of EMF. The field of bioelectromagnetic research is still mainly focused on the initial exploration of biological effects. Accordingly, many countries are continuously studying the possible biological effects of various EMF sources and effective protection measures in addition to the application of EMF [4–6].

According to a meta-analysis on the effects of EMF exposure on human health, the International Commission on Non-Ionizing Radiation Protection (ICNIRP) and the Institute of Electrical and Electronics Engineers (IEEE) individually declared more stringent guidelines for exposures to EMF from 0 direct current (DC) to 300.0 GHz (IEEE, 2019; ICNIRP, 2020).

As we all know, the physical properties of EMFs are closely related to their frequency. The key parameters of EMF, such as reflectivity, penetration, and absorptivity, vary with its frequency. Therefore, EMFs with different frequencies are defined as multiple bands for particular purposes based on the above characteristics. Over the last 20 years, researchers have compiled increasingly strong evidence that EMFs over the entire frequency range can modify biological processes. There is now solid experimental evidence and a theoretical basis indicating that weak EMFs, especially but not exclusively EMFs at low frequencies, can cause symptoms such as irritability, headache, memory loss, and increased incidence of brain tumors [7, 8]. A large number of animal experiments have also found that electromagnetic radiation with certain parameters can reduce learning ability and memory, affect emotions, and impair the brain structure and function [9–13].

The frequency band of the EMF used in this study was centered around 2.0 GHz, corresponding to the L-band EMF that is widely used in satellite navigation systems. Due to the complexity of operating this kind of navigation system, the operators need to have higher cognitive ability. If the operators have neurocognitive dysfunction, such as difficulty concentrating, slow reaction, and impaired ability to read, it can easily lead to adverse consequences. Therefore, it is critical to investigate whether L-band EMF has an adverse effect on brain function.

According to the different mechanisms through which an EMF exerts its effects on organisms, they can be divided into thermal effects and nonthermal effects. When a biological system is exposed to high-frequency electromagnetic radiation, the heat generated by molecular movement cannot be released in a short time, leading to the thermal effects under the action of dipoles. Furthermore, the balance of the weak EMF of the organism can be disturbed, resulting in nonthermal effects after long-term exposure to low-frequency electromagnetic radiation. If the damage caused by thermal and nonthermal effects cannot be fully repaired, it will accumulate after renewed exposure to EMF radiation [14]. Consequently, diseases might be induced by long-term exposure to EMF because of the accumulation effect, which should be investigated with great care [15]. It has been reported that a thermal effect is induced when the power density of EMF exceeds 100 W/m^2^. However, microthermal effects are dominant at power densities of 10–100 W/m^2^ and nonthermal effects are dominant at power densities of less than 10 W/m^2^ [16]. In the present study, a central frequency of 2.0 GHz and a maximum power density of 1.5 W/m^2^ were adopted. Therefore, it can be assumed based on previous results that the treatment with L-band electromagnetic radiations in this study mainly induced nonthermal effects.

There is a weak but stable EMF in the human body, and EMF of a certain intensity could interfere with the bioelectrical activities of the human body and make it unstable. Since the functioning of the nervous system is based on bioelectricity, it is more susceptible to be influenced by external EMF. The nervous system is believed to be an important and sensitive target for electromagnetic exposure. A variety of damaging effects at the whole body, tissue, cell, and gene levels would be induced by long-term exposure to EMF [17–19]. Therefore, to explore the effects of continuous long-term exposure to L-band EMF on brain function and structure, EMFs with an average power density of 0.5, 1.0, and 1.5 W/m^2^ were used to irradiate Institute of Cancer Research (ICR) mice for 4 or 8 weeks in the present study. This research provides a biomedical reference for operators of the L-band EMF platform.

## 2. Materials and Methods

### 2.1. Animals

All animal care and experimental procedures were in accordance with the University Policies on the Use and Care of Animals and were approved by the Institutional Animal Experiment Committee of Air Force Medical University, Xi'an, China (identification code: IACUC-20180503; date of approval: 21 May 2018). A total of 48 male ICR mice (5–6 weeks old, weighing 18 ± 2 g) were obtained from the Laboratory Animal Center of Air Force Medical University and housed in ten different cages in temperature- and humidity-controlled rooms with ad libitum access to food and water throughout the experimental period.

### 2.2. L-HPM Exposure Protocol and Experimental Groups

The L-HPM exposure was carried out in a microwave anechoic chamber which included a shielding room and a control room. The shielded enclosure was made of steel plates and had an inner wall covered with a tapered carbon sponge absorbing material to shield from interference by external electromagnetic fields. The L-HPM exposure facility was placed in the shielded room. Animals were placed in a special plastic box in a free position on the animal platform.

The 48 male ICR mice were randomly divided into four groups: sham exposure group, 0.5 W/m^2^ L-HPM exposure group, 1.0 W/m^2^ L-HPM exposure group, and 1.5 W/m^2^ L-HPM exposure group, with 12 mice per group. According to the different exposure times and sampling times, mice from each group were randomly divided into two subgroups with six animals in each subgroup. Animals in the first subgroup were sham exposed or received whole-body exposure to L-HPM for 4 weeks (1 h/day, 09:00 am–10:00 am) and sacrificed 2 weeks after exposure. Animals in the second subgroup were sham exposed or received whole-body exposure to L-HPM for 8 weeks (1 h/day, 09:00 am–10:00 am) and sacrificed 6 weeks after exposure.

### 2.3. Preparation of Brain Tissue for Analysis

After L-HPM sham exposure or exposure, the whole brains of mice were removed. Three brains from each subgroup of six animals were fixed in 4% paraformaldehyde for 24 h and embedded in paraffin following standard methods for immunohistochemical and histological analyses. The other three brains were quickly dissected on ice and immediately snap frozen and stored at −80°C to be used for the biochemical analyses.

### 2.4. Hematoxylin and Eosin (HE) Staining

The whole brains were isolated and fixed in 4% paraformaldehyde for 24 h and then rinsed in running water for 24 h. Thereafter, each sample was dehydrated using a standard alcohol series, defatted in xylene, and finally embedded in paraffin. Histological sections, 5 *μ*m thick, were dewaxed, hydrated, and stained with HE. After drying, the histological slides were covered with cover slips. Digital photography of the hippocampus and cerebral cortex was performed under a Leica DMI4000B optical microscope (Leica Biosystems, Heidelberg, Germany) and a Hamamatsu NanoZoomer Scan SQ1.0 (Hamamatsu Photonics, Shizuoka, Japan) with NDP.

### 2.5. TUNEL Staining

Apoptotic cells in the brains were identified via the terminal deoxynucleotidyl transferase-mediated dUTP nick-end labeling (TUNEL) assay using the in situ Cell Death Detection Kit (Roche, Germany) according to the manufacturer's protocol. Briefly, the brain sections were deparaffinized before rehydration with decreasing concentrations of ethanol. Subsequently, the sections were washed with PBS pH 7.4 and then covered with proteinase K solution for 25 min. Thereafter, the sections were washed with PBS again, covered with the TUNEL reaction mixture, and incubated for 1 h in the dark. DAPI counterstaining of nuclei was followed by a final PBS wash. Florescence micrographs were obtained using a Nikon Eclipse C1 fluorescence microscope (Nikon, Japan) and analyzed using CaseViewer software. The number of TUNEL-positive cells were counted under 400-fold magnification. Cell counting was performed by an investigator blinded to the groups.

### 2.6. Immunohistochemistry

The brains were cut in the coronal plane into sections with a thickness of 5 *μ*m, which were mounted on slides. Sections at the level of the hippocampus and cerebral cortex were deparaffinized and rehydrated via a decreasing alcohol gradient. Endogenous peroxidase activity was quenched using 3% hydrogen peroxide in methanol for 30 min in darkness, and 0.01 M citrate buffer pH 6.0 was applied for microwave antigen regeneration. The brain sections were washed with PBS and incubated with blocking solution for 1 h at room temperature. Then, the slides were incubated with rabbit anti-c-fos primary antibody (1 : 1000, Servicebio, China) at 4°C overnight. After washing in PBS, a secondary goat anti-rabbit antibody conjugated with horseradish peroxidase (HRP) (1 : 200, Servicebio, China) was incubated with the slides for 1 h at room temperature. Thereafter, the color was developed using DAB (BosterBio, USA). Following hematoxylin counterstaining, slides were sealed with neutral gum. The sections were observed and photographed using a conventional optical microscope (Nikon, Japan). Finally, the positive cells in the hippocampus and cerebral cortex from each mouse were counted at 400-fold magnification in five randomly chosen visual fields.

### 2.7. C-fos Expression Analysis

Image-Pro Plus 6.0 software was used to analyze the positive cumulative optical density of each image (IOD) and the pixel area of the tissue (AREA). Average optical density (AO) was calculated as IOD/AREA; this AO value was proportional to the positive expression level.

### 2.8. Assay of Cholinergic Markers

Cholinergic marker levels in brain tissue were measured using commercial assay kits (Nanjing Jiancheng Bioengineering Institute, China). Brain tissue was weighed and homogenized in 9 volumes of ice-cold saline containing a protease inhibitor cocktail (Sigma-Aldrich) and centrifuged at 3000 rpm and 4°C for 20 min to obtain the cleared lysate. The total protein concentration was quantified by the Bradford assay method using the Bio-Rad Dc System (Bio-Rad Laboratories, USA). The cleared lysate was further diluted with the appropriate buffer solutions to measure the activities of choline acetyltransferase (ChAT) and acetylcholinesterase (AChE), according to the manufacturer's instructions. All samples were assessed in triplicate.

### 2.9. Determination of Oxidative Stress Marker Levels

Oxidative stress marker levels in brain tissue were measured using commercially available assay kits (Nanjing Jiancheng Bioengineering Institute, China). Cleared brain tissue lysate obtained as described in Section 2.8 was used to measure the activities of superoxide dismutase (SOD) and the content of malondialdehyde (MDA) spectrophotometrically using assay kits according to the manufacturer's instructions. All samples were measured in triplicate.

### 2.10. Statistical Analysis

All data were presented as means ± standard deviations (SD) and analyzed using SPSS 22.0 software (SPSS Inc., USA). Student's *t*-test followed by homogeneity of the variance test was used to analyze the significance of differences between the sham-exposed group and exposed group. For all statistical analyses, a *P* value < 0.05 was considered to indicate statistical significance.

## 3. Results

### 3.1. Effect of L-HPM Exposure on the Morphology of the Hippocampus and Cerebral Cortex

**Figure 1:**
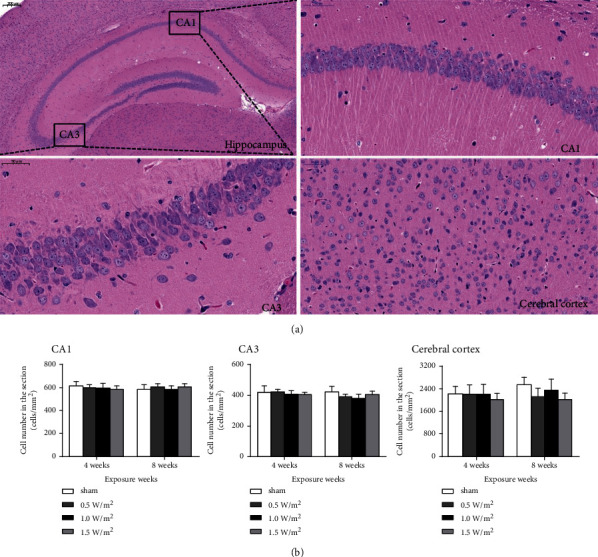
HE stained sections of the mouse hippocampus (CA1 and CA3) and cerebral cortex (S1) after L-HPM exposure for different durations. (a) The histology of the hippocampus and cerebral cortex examined by HE staining. (b) Morphometric analysis of the hippocampus and cerebral cortex. Scale bar = 50 *μ*m. HE: hematoxylin-eosin; L-HPM: L-band high-power microwave.

The brain sections stained with HE did not show any significant morphological and morphometric differences in the hippocampus (CA1 and CA3) and cerebral cortex (the first somatosensory cortex, S1) between the L-HPM-exposed group and the sham-exposure group (Figure 1).

### 3.2. L-HPM Exposure Aggravated the Cell Apoptosis in the Hippocampus and Cerebral Cortex

**Figure 2:**
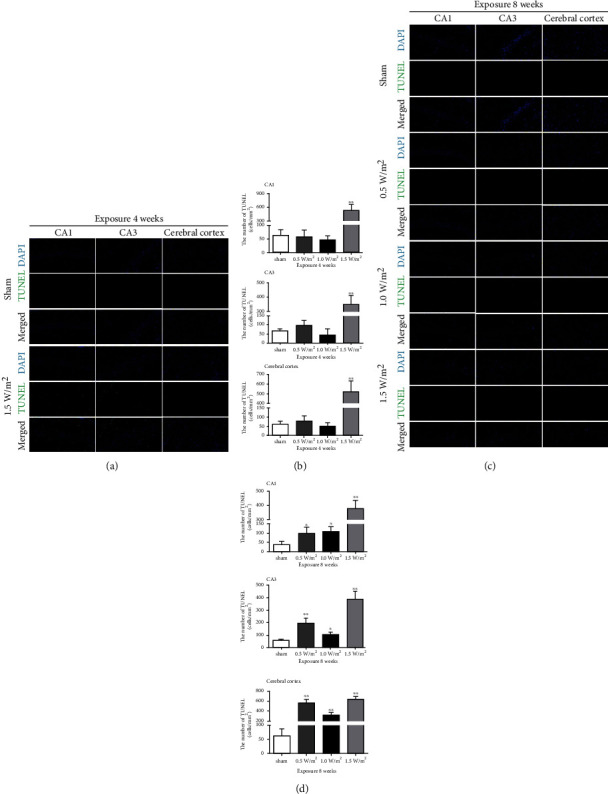
TUNEL staining of the hippocampus (CA1 and CA3) and cerebral cortex (S1) of mice after L-HPM exposure for different durations. Original magnification: ×400. (a) Representative image of TUNEL staining of the hippocampus and cerebral cortex after 4 weeks of L-HPM exposure (scale bar = 50 *μ*m). (b) Quantification of TUNEL-positive cells/mm^2^ in the hippocampus and cerebral cortex after 4 weeks of L-HPM exposure. (c) Representative image of TUNEL staining of the hippocampus and cerebral cortex after 8 weeks of L-HPM exposure (scale bar = 50 *μ*m). (d) Quantification of TUNEL-positive cells/mm^2^ in the hippocampus and cerebral cortex after 8 weeks of L-HPM exposure. Student's *t*-test, mean ± SEM, ^∗^*P* < 0.05, ^∗∗^*P* < 0.01 compared with the sham group. TUNEL: deoxynucleotidyl transferase-mediated dUTP nick-end labeling; L-HPM: L-band high-power microwave.

To assess the effects of L-HPM exposure on cell apoptosis, TUNEL staining was performed. In the group exposed for 4 weeks, the mice exposed to L-HPM with an intensity of 1.5 W/m^2^ showed a significantly increased number of TUNEL-positive cells in the hippocampal CA1 and CA3 and cerebral cortex S1 (Figures 2(a) and 2(b)) than those in the sham exposure group. In the group exposed for 8 weeks, the mice exposed to L-HPM with intensities of 0.5, 1.0, and 1.5 W/m^2^ showed a significant increase in the number of TUNEL-positive cells compared with those in the sham exposure group (Figures 2(c) and 2(d)). These results demonstrated that L-HPM exposure may aggravate the apoptosis of neurons and glial cells in the hippocampus and cerebral cortex.

### 3.3. Effect of L-HPM Exposure on c-fos Levels in the Mouse hippocampus and Cerebral Cortex

**Figure 3:**
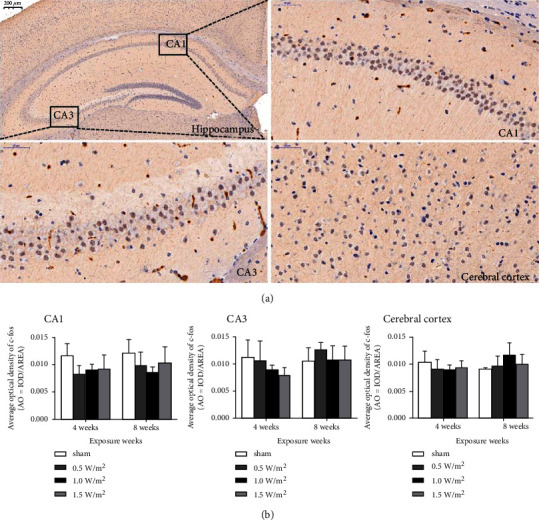
Distribution (a) and expression (b) of c-fos in the mouse hippocampus (CA1 and CA3) and cerebral cortex (S1) after L-HPM exposure for different durations (original magnification: ×400, scale bar = 50 *μ*m). L-HPM: L-band high-power microwave.

Immunohistochemical staining was used to evaluate the distribution and expression level of c-fos. The results showed that the distribution of c-fos did not change following L-HPM exposure for different durations (Figure 3(a)). Quantitative analysis revealed that the expression level of c-fos in the hippocampal CA1 and CA3 and cerebral cortex S1 did not exhibit significant differences between the L-HPM-exposed groups and the sham exposure group (Figure 3(b)).

### 3.4. Effects of L-HPM Exposure on the Activity of AChE and ChAT in the Mouse Brain

**Figure 4:**
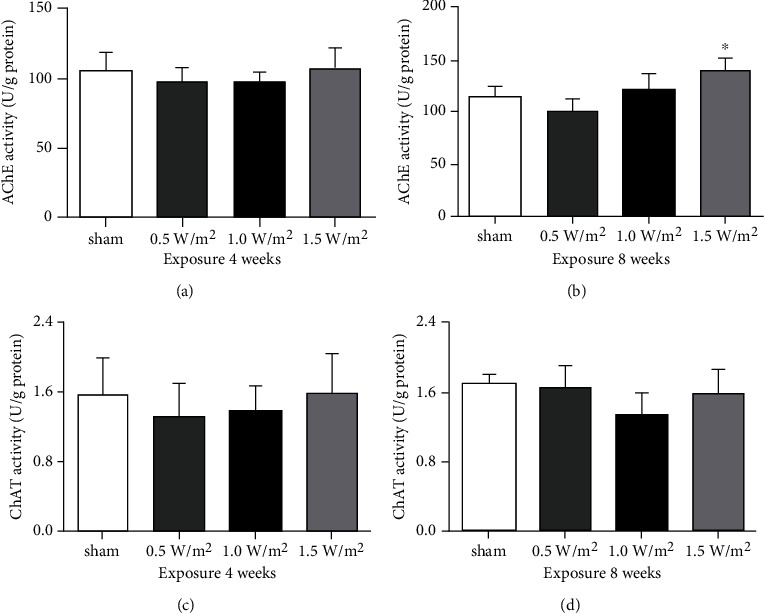
The activity of AChE (a, b) and ChAT (c, d) in mouse brains after L-HPM exposure for different durations. Student's *t*-test, mean ± SEM, ^∗^*P* < 0.05 compared with the sham group. AChE: acetylcholinesterase; ChAT: choline acetyltransferase.

Biochemical analyses revealed that the activity of AChE was significantly increased in the mouse brain after 8 weeks of exposure to L-HPM at 1.5 W/m^2^ (Figure 4(b)). These results indicate that long-term high-dose L-HPM exposure may cause central cholinergic dysfunction. However, no differences in ChAT and AChE activity were observed between the groups exposed to L-HPM at 0.5 or 1.0 W/m^2^ and the sham exposure group (Figures 4(a), 4(c), and 4(d)).

### 3.5. Effects of L-HPM Exposure on the Activity of SOD and the Content of MDA in the Mouse Brain

**Figure 5:**
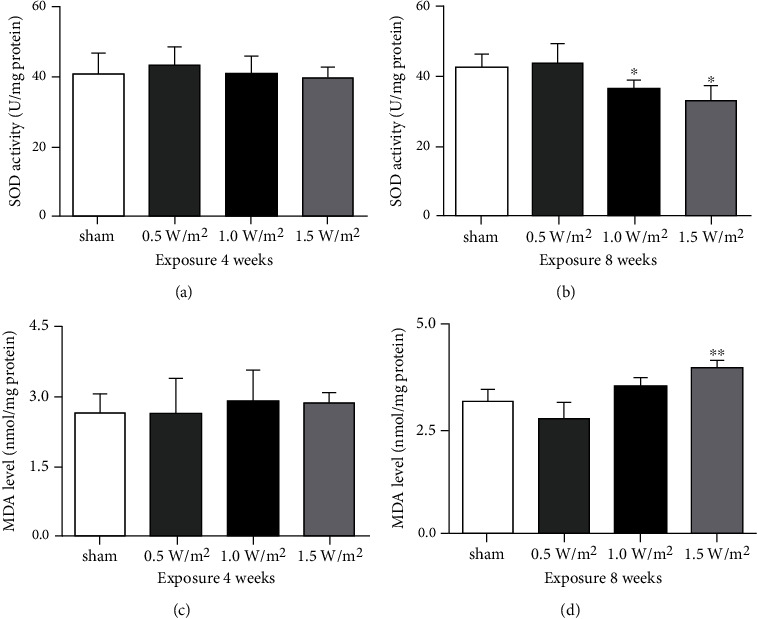
The activity of SOD (a, b) and the content of MDA (c, d) in mouse brains after L-HPM exposure for different durations. Student's *t*-test mean ± SEM, ^∗^*P* < 0.05, ^∗∗^*P* < 0.01 compared with the sham group.

Biochemical analyses showed that the activity of SOD in the mouse brain was significantly decreased after 8 weeks of exposure to L-HPM at 1.0 and 1.5 W/m^2^ exposure (Figure 5(b)). Furthermore, the MDA levels were significantly elevated after 8 weeks of exposure to L-HPM at 1.5 W/m^2^ (Figure 5(d)). However, there were no significant differences in SOD and MDA levels between the other L-HPM exposure groups and the sham exposure group (Figures 5(a) and 5(c)). This indicates that increased oxidative damage was induced by long-term high-dose L-HPM exposure, while shorter and low-dose L-HPM exposure did not induce oxidative damage.

## 4. Discussion

The major finding of the present study is that the exposure to L-HPM at 1.5 W/m^2^ can cause generalized injuries in the central nervous system of mice, including cell apoptosis, cholinergic dysfunction, and oxidative damage. Our results demonstrated that the damaging effects are closely related to the power density and exposure time, indicating that long-term L-HPM exposure at high power densities may be detrimental to human health, especially the nervous system.

The cerebral cortex and hippocampus CA1 and CA3 regions are considered to be vulnerable areas of the brain [20], in which selective cell damage or loss is closely related to cognitive impairment [12, 21]. In order to assess possible changes in the morphological structure of the treated mice, we analyzed the morphological structure and cell number in the hippocampus and cerebral cortex after L-HPM exposure. The results of HE staining suggested that the morphological structure of the hippocampal CA1 and CA3 and cerebral cortex S1 did not change in the exposed groups compared with that in the sham exposure group. Next, we examined the effects of L-HPM exposure on the number of hippocampal and cortical cells. Results of TUNEL staining revealed that cell apoptosis in the cerebral cortex S1 and hippocampus CA1 and CA3 regions of mice in the exposed groups significantly increased as compared to those in the sham exposure group. Furthermore, the percentage of apoptotic cells significantly increased in the groups exposed to L-HPM at 1.5 W/m^2^ compared to 0.5 and 1.0 W/m^2^. Consistent with previous observations, our findings supported the idea that L-HPM exposure could induce neuron and glial cell apoptosis in mice, indicating that it could cause changes in the cognitive function of mice. Our results also confirmed that the degree of reduction in the number of neurons and glial cells was closely related to the power density, which means that the dose-effect relationship of exposure is essential for its safety assessment.

Several studies have shown that c-fos is a major stress-related protein which can be induced by various stimuli such as injury, heat, and exposure to electromagnetic radiation [22–24]. C-fos protein expression in cells is very low and is not significantly affected by stimulation without injury. To some extent, the number of c-fos-positive cells is directly proportional to the intensity of stimulation and the upregulation of c-fos expression often indicates that cells are exposed to noxious stimuli [25]. Thus, the expression levels of c-fos in the cerebral cortex S1 as well as the hippocampal CA1 and CA3 regions were observed to evaluate the effect of L-HPM on neurons and glial cells in mice. Our results showed that there was no significant difference in c-fos protein expression in the hippocampus and cerebral cortex between the exposed groups and the control group.

Although L-HPM exposure could cause cell apoptosis in the cerebral cortex and hippocampus, which may lead to cognitive deficits in mice, the underlying mechanisms are not fully understood. ChAT and AChE are closely related to the metabolism of acetylcholine, a neurotransmitter that plays an important role in learning and memory modulation [26, 27]. In addition, studies have shown that exposure to electromagnetic radiation could decrease ChAT activity and increase AChE activity in the brain, which in turn affects the functioning of the nervous system [13]. Consistent with previous studies, here, we found a marked increase in AChE activity in the brains of mice exposed to L-HPM at 1.5 W/m^2^ for 8 weeks. Thus, our results suggested that the destructive effect of L-HPM exposure may stem from reduced central cholinergic function due to the inhibition of the synthesis and release of acetylcholine. Furthermore, recent researches have revealed that central cholinergic dysfunction is related to increased oxidative stress.

SOD is an effective scavenger of free radicals and is one of the most important antioxidative enzymes in the body. MDA is an indicator of lipid peroxidation due to oxidative stress. As oxidative stress is an imbalance between the production of cell-damaging free radicals and the body's ability to neutralize them, in conditions of oxidative stress, SOD activity is usually decreased, while the MDA content is increased. Previous studies have demonstrated that oxidative damage is directly correlated with exposure-induced brain disorders [19, 28, 29]. In our study, we further confirmed that L-HPM exposure could induce oxidative damage, as shown by significantly decreased activity of SOD and increase of lipid peroxidation (MDA) in the brains of mice exposed to L-HPM at 1.5 W/m^2^ for 8 weeks. These results therefore indicate that long-term high-intensity L-HPM exposure may substantially increase oxidative damage in the brain.

Several limitations should be considered when interpreting this study. Firstly, we did not perform behavioral tests on the mice, because the purpose of the experiment was to clarify whether L-HPM would lead to nervous system damage, and we did not focus on cognitive function. Nevertheless, further studies should include behavioral tests on animals after L-HPM exposure. Secondly, other potential molecular mechanisms leading to cell apoptosis after exposure to L-HPM remain to be further studied.

## 5. Conclusions

Taken together, the results of this study demonstrate that L-HPM exposure at certain power densities can lead to oxidative stress in hippocampal and cortical cells and induce brain injury in mice.

## Data Availability

The authors confirm that all the data supporting the findings of this study are available within the article.

## References

[1] Li J. H., Jiang D. P., Wang Y. F. (2017). Influence of electromagnetic pulse on the offspring sex ratio of male BALB/c mice. *Environmental Toxicology and Pharmacology*.

[2] Narayanan S. N., Jetti R., Kesari K. K., Kumar R. S., Nayak S. B., Bhat P. G. (2019). Radiofrequency electromagnetic radiation-induced behavioral changes and their possible basis. *Environemental Science and Pollution Research*.

[3] Shahin S., Banerjee S., Swarup V., Singh S. P., Chaturvedi C. M. (2018). From the cover: 2.45-GHz Microwave radiation impairs hippocampal learning and spatial memory: involvement of local stress mechanism-induced suppression of iGluR/ERK/CREB signaling. *Toxicological Sciences*.

[4] Romanenko S., Begley R., Harvey A. R., Hool L., Wallace V. P. (2017). The interaction between electromagnetic fields at megahertz, gigahertz and terahertz frequencies with cells, tissues and organisms: risks and potential. *Journal of The Royal Society Interface*.

[5] Sultangaliyeva I., Beisenova R., Tazitdinova R., Abzhalelov A., Khanturin M. (2020). The influence of electromagnetic radiation of cell phones on the behavior of animals. *Veterinary World*.

[6] Turner M. C., Benke G., Bowman J. D. (2017). Interactions between occupational exposure to extremely low frequency magnetic fields and chemicals for brain tumour risk in the INTEROCC study. *Occupational and Environmental Medicine*.

[7] Speers M. A., Dobbins J. G., Miller V. S. (1988). Occupational exposures and brain cancer mortality: a preliminary study of East Texas residents. *American Journal of Industrial Medicine*.

[8] Calvente I., Perez-Lobato R., Nunez M. I. (2016). Does exposure to environmental radiofrequency electromagnetic fields cause cognitive and behavioral effects in 10-year-old boys?. *Bioelectromagnetics*.

[9] Li H. J., Peng R. Y., Wang C. Z. (2015). Alterations of cognitive function and 5-HT system in rats after long term microwave exposure. *Physiology & Behavior*.

[10] Di G., Kim H., Xu Y., Kim J., Gu X. (2019). A comparative study on influences of static electric field and power frequency electric field on cognition in mice. *Environmental Toxicology and Pharmacology*.

[11] Shahin S., Banerjee S., Singh S. P., Chaturvedi C. M. (2015). 2.45 GHz microwave radiation impairs learning and spatial memory via oxidative/nitrosative stress induced p53-dependent/independent hippocampal apoptosis: molecular basis and underlying mechanism. *Toxicological Sciences*.

[12] Tan S., Wang H., Xu X. (2017). Study on dose-dependent, frequency-dependent, and accumulative effects of 1.5 GHz and 2.856 GHz microwave on cognitive functions in Wistar rats. *Scientific Reports*.

[13] Zhang J. P., Zhang K. Y., Guo L. (2017). Effects of 1.8 GHz radiofrequency fields on the emotional behavior and spatial memory of adolescent mice. *International Journal of Environmental Research and Public Health*.

[14] Fields E., Cleveland R. F., Ulcek J. L. (1999). Questions and answers about biological effects and potential hazards of radiofrequency electromagnetic fields. *OET Bulletin*.

[15] Barnes F., Greenebaum B. (2020). Setting guidelines for electromagnetic exposures and research needs. *Bioelectromagnetics*.

[16] Frohlich H. (1975). The extraordinary dielectric properties of biological materials and the action of enzymes. *Proceedings of the National Academy of Sciences of the United States of America*.

[17] Deshmukh P. S., Megha K., Nasare N. (2016). Effect of low level subchronic microwave radiation on rat brain. *Biomedical and Environmental Sciences*.

[18] Narayanan S. N., Mohapatra N., John P. (2018). Radiofrequency electromagnetic radiation exposure effects on amygdala morphology, place preference behavior and brain caspase-3 activity in rats. *Environmental Toxicology and Pharmacology*.

[19] Sharma S., Shukla S. (2020). Effect of electromagnetic radiation on redox status, acetylcholine esterase activity and cellular damage contributing to the diminution of the brain working memory in rats. *Journal of Chemical Neuroanatomy*.

[20] Li E., Kim D. H., Cai M. (2011). Hippocampus-dependent spatial learning and memory are impaired in growth hormone-deficient spontaneous dwarf rats. *Endocrine Journal*.

[21] Gupta S. K., Mesharam M. K., Krishnamurthy S. (2018). Electromagnetic radiation 2450 MHz exposure causes cognition deficit with mitochondrial dysfunction and activation of intrinsic pathway of apoptosis in rats. *Journal of Biosciences*.

[22] Herrera D., Robertson H. (1996). Activation of *c-fos* in the brain. *Progress in Neurobiology*.

[23] Otsuka M., Hatakenaka M., Ishigami K., Masuda K. (2001). Expression of the c-*myc* and c-*fos* genes as a potential indicator of late radiation damage to the kidney. *International Journal of Radiation Oncology • Biology • Physics*.

[24] Velazquez F. N., Caputto B. L., Boussin F. D. (2015). c-Fos importance for brain development. *Aging*.

[25] Bullitt E. (1990). Expression of c-fos-like protein as a marker for neuronal activity following noxious stimulation in the rat. *The Journal of Comparative Neurology*.

[26] Haam J., Yakel J. L. (2017). Cholinergic modulation of the hippocampal region and memory function. *Journal of Neurochemistry*.

[27] Hampel H., Mesulam M. M., Cuello A. C. (2018). The cholinergic system in the pathophysiology and treatment of Alzheimer’s disease. *Brain*.

[28] Liu C., Duan W., Xu S. (2013). Exposure to 1800 MHz radiofrequency electromagnetic radiation induces oxidative DNA base damage in a mouse spermatocyte-derived cell line. *Toxicology Letters*.

[29] Lin Y. Y., Wu T., Liu J. Y. (2018). 1950MHz radio frequency electromagnetic radiation inhibits testosterone secretion of mouse Leydig cells. *International Journal of Environmental Research and Public Health*.

